# Supplementary material for: Concurrence of anemia and stunting and associated factors among children aged 6 to 59 months in Peru
Source: PLOS Glob Public Health. 2024 Apr 2;4(4):e0002914. doi: 10.1371/journal.pgph.0002914 (PMC10986945; doi:10.1371/journal.pgph.0002914)
Supplement: S1 Table — (DOCX) [file pgph.0002914.s001.docx]

| **Variables** | **Variable type and categories** | **Original survey question and/or description** |
| --- | --- | --- |
| ***Basic factors (distal)*** | | |
| Residence | Binary categorized as urban and rural. | This is the geographic area to which the respondent's home belongs, this area can be urban or rural. |
| Mother currently working | Binary categorized as yes and no. | Last week, between Sunday ________ and Saturday |
| Wealth index | Qualitative ordinal with 5 categories: poorest, poorer, middle, wealthier and wealthiest. | Is a composite measure of a household's cumulative living standard. The wealth index is calculated using data on a household's ownership of selected assets, such as televisions and bicycles; materials used for housing construction; and types of water access and sanitation facilities. |
| Maternal marital status | Binary categorized as united and not united. | Are you currently united or cohabiting?  It was recategorized into unmarried (married and living together) and not united (never married, widowed, divorced, not living together). |
| Maternal insurance coverage | Binary categorized as yes and no. | Women who have some type of insurance (social insurance, armed or police forces, public health insurance, health provider, other) or none. |
| Maternal education level | Qualitative ordinal. | What was the highest level of education you passed? Recategorized as no formal education/primary, secondary and higher. |
| Paternal education level | Qualitative ordinal. | What was the highest level of education you passed? Recategorized as no formal education/primary, secondary and higher. |
| Maternal ethnic group membership | Binary categorized as yes and no. | What language or dialect do they usually speak at home?  Re-categorized as "No" if the woman speaks Spanish, Portuguese or English. It was recategorized as "Yes" in the other cases according to the Database of Indigenous or Original Peoples of the Ministry of Culture - Peru. |
| Natural region | Qualitative nominal. Categorized as metropolitan Lima, rest of the coast, highlands, and jungle. | The variable allows us to know the natural region where the survey was conducted. |
| ***Underlying factors (intermediate)*** | | |
| Water source | Binary categorized as improved and unimproved. | What is the main source of water supply used in your household for drinking?  Improved category [inside the dwelling, outside the dwelling but inside the building, public pylon/tap, well in dwelling/yard/lot, spring (puquio), rainwater, tanker truck, and bottled water], and unimproved [public well, river/waterhole/lagoon, and other] according to the DHS guide. |
| Household appliances | Binary categorized into owns and does not own. | Do you have in your home: Equipment  a. radio? b. television? c. refrigerator/freezer?  Evaluated as own if there was an affirmative answer to at least 1 household appliances. |
| Transportation | Binary categorized into owns and does not own | Does any member of your household have:  a. bicycle/tricycle?  b. motorcycle / motorcycle cart?  c. car or truck?  Evaluated as own if there was an affirmative answer to at least 1 transportation. |
| Flooring material | Binary categorized as improved and unimproved. | Predominant housing flooring material (by observation).  The categories were improved [wood (decking), parquet or polished wood, asphalt sheeting, vinyl or similar, tile, terrazzo or similar, and cement/brick], and unimproved [earth/sand, pona, other (pona)] according to the DHS guide. |
| Walling material | Binary categorized as improved and unimproved. | Predominant material of the exterior walls of the dwelling (by observation).  The categories were improved [planks/wood, adobe or tapia, quincha, stone with mud, brick or cement blocks, stone or ashlar with lime or cement, tripley], and unimproved [mat, cane/bamboo/pona/palm/logs/tabique, cardboard, no walls, other] according to the DHS guide. |
| Roofing material | Binary categorized as improved and unimproved. | Predominant roofing material of the dwelling (by observation).  The categories were improved (reinforced concrete, wood, roof tiles, calamine sheet, fiber cement or similar), and unimproved (thatch, palm leaves, etc., mat, reed or mat with mud cake, cardboard, no roof, other) according to the DHS guide. |
| ***Proximal factors (immediate)*** | | |
| Sex | Categorized as female or male. | Is (NAME) male or female?  Sex of each child of the respondent. |
| Child age | Qualitative ordinal. Categorized into 6-11 months, 12-13 months, 24-35 months, 36-47 months and 48-59 months. | How old is (NAME)? |
| Birthweight | Qualitative ordinal. Categorized into underweight (< 2500 g), normal (2500 - 4000 g) and macrosomic (> 4000 g). | How much did (NAME) weigh? |
| Delivery | Binary categorized as single or multiple. | Was (NAME)'s birth multiple or single birth? |
| Cesarean delivery | Binary categorized as yes and no. | (NAME) Was (NAME) born by cesarean section? |
| Minimum dietary diversity | Assessed by whether the child received 24 complementary foods from the 7 food groups in the previous 24 hours. Recategorized as yes and no. | Defined as "yes" according to DHS guidelines as consumption of at least 4 of 7 food groups during the day or night prior to the survey:  (a) Grains, roots and tubers, (b) Legumes and nuts, (c) Dairy products (milk, yogurt, cheese), (d) Meat foods (meat, fish, poultry and liver/vegetables), (e) Eggs, (f) Fruits and vegetables rich in vitamin A, (g) Other fruits and vegetables.  Otherwise, it was recategorized as "no". |
| Immediate breastfeeding | Binary categorized as yes and no. | How long after (NAME) was born did you start breastfeeding?  Defined as “yes” is breastfeeding was within 60 minutes of delivery. |
