# Supplementary material for: Concurrence of anemia and stunting and associated factors among children aged 6 to 59 months in Peru
Source: PLOS Glob Public Health. 2024 Apr 2;4(4):e0002914. doi: 10.1371/journal.pgph.0002914 (PMC10986945; doi:10.1371/journal.pgph.0002914)
Supplement: Attachment — Submitted filename: Review report.docx [file pgph.0002914.s003.docx]

**Review report**

Title: Concurrence of anemia and stunting in children aged 6 to 59 months and its associated factors in Peru

Dear Editor:

Thank you very much for inviting me to review an article entitled “Concurrence of anemia and stunting in children aged 6 to 59 months and its associated factors in Peru”.

The study is interesting in its field considering the magnitude of the problem globally, and the study fills the knowledge gap on concurrent anemia and stunting in the country by using a nationally representative data.

The paper is well written. However, the authors need to consider the following **major and minor** concern substantially.

**Abstract section**

1. Line 18 -19 – the authors stated “The analysis design was cross-sectional….... better to say *study design*….
2. Procedures for anthropometry and hemoglobin measurement were not described in the abstract section. Pleas describe briefly.

**Background**

3. The paper provides very limited background information particularly with regards to the efforts made by concerned body to address the issue and the research gap they have intended to fill.

**Methods**

4. It would be interesting to have some details of the respondents: who were selected to be the responsible for the answers; were the respondents only mothers or any person present at the household could answer the questionnaire? What specific criteria were used to select the respondents?
5. The authors stated that "Variables with P<0.10 value in the bivariate analyses were included in the multivariate analyses. Thus, model-1 included the distal factors which demonstrated P<0.10". Did you consider the theoretical model in the inclusion of variables? It is quite important to understand that the theoretical model should be above statistical significance.
6. The authors built single construct of Concurrent Anemia and Stunting. Have you thought of considering testing a construct of determination of the outcome divided in four categories: only stunting, only anemia, CAS versus none? It would have been interesting to analyze and compare the single odds ratio of each outcome and both outcome to verify if the odds are higher when they are combined then only anemia or stunting.
