# Supplementary material for: Concurrence of anemia and stunting and associated factors among children aged 6 to 59 months in Peru
Source: PLOS Glob Public Health. 2024 Apr 2;4(4):e0002914. doi: 10.1371/journal.pgph.0002914 (PMC10986945; doi:10.1371/journal.pgph.0002914)
Supplement: Attachment — Submitted filename: Response to Reviewers.docx [file pgph.0002914.s004.docx]

**Response to Reviewers**

**Comments to the Author**

We would like to thank everyone for their constructive comments in this round of review. Your comments have provided valuable insights to refine the content and analysis of our article. In this paper we try to address the comments raised in the best way.

**Reviewer #1:**

**Abstract**

1. **Line 18 -19 – the authors stated “The analysis design was cross-sectional….... better to say study design….**

**Response**:

a. Thank you for this observation. We corrected as suggested by reviewer.
2. **Procedures for anthropometry and hemoglobin measurement were not described in the abstract section. Please describe briefly.**

**Response**:

a. Thank you for this comment. We added the following text in the abstract:

“To reduce error in measures, the anthropometry personnel was training, the quality of measuring equipment was ensuring, and protocolized techniques and procedures was applying. Hemoglobin concentration was measured in capillary blood using the Hemocue model Hb 201+.”

**Background**

3. **The paper provides very limited background information particularly with regards to the efforts made by concerned body to address the issue and the research gap they have intended to fill.**

**Response:**

a. Thank you for this comment. In order to identify the research gap clearly, we add the following text (line 79 – 85): “Among the objectives of UNICEF's Nutrition Strategy for 2020-2023 is to prevent undernutrition, micronutrient deficiencies and overweight during the first five years of life. While UNICEF has a conceptual framework for preventing all forms of malnutrition, it does not identify specific risk or protective factors for the concurrent anemia and stunting (CAS). The absence of a specific conceptual framework for this concurrence is because there are few studies that have explored the factors associated with the CAS. To our knowledge, factors associated with the CAS have not been examined in a nationally representative sample of children under five years of age in Peru.

**Methods**

4. **It would be interesting to have some details of the respondents: who were selected to be the responsible for the answers; were the respondents only mothers or any person present at the household could answer the questionnaire? What specific criteria were used to select the respondents?**

**Response:**

a. Thank you for this comment. The responsible for give the answers to the survey questions are the mothers. We consider that this information is present in the third paragraph of section “study design”. The inclusion criteria are described in the second paragraph of section “setting and participants”. Also, we add that “The DHS conducts interviews with all women in the selected household”.
5. **The authors stated that "Variables with P<0.10 value in the bivariate analyses were included in the multivariate analyses. Thus, model-1 included the distal factors which demonstrated P<0.10". Did you consider the theoretical model in the inclusion of variables? It is quite important to understand that the theoretical model should be above statistical significance.**

**Response:**

a. Thanks for the comment. We agree with the reviewer. The third paragraph of section “statistical analysis” describe the process of including the variables in the regression model.

“For the multivariate analysis we applied a three-level binary hierarchical logistic regression model. The conceptual framework of stunting and CAS supports the use of this analytical approach, as the explanatory variables are configured into distal, intermediate, and immediate factors. The adaptation of a hierarchical model to study the determinants of CAS is based on the recommendations of Victora et al [24]. The epidemiological criterion allowed the identification of the explanatory variables, then based on a statistical criterion, those variables with a p value < 0.10 in the crude analysis were included for the multivariate analysis.”

The theoretical model that we followed was based on UNICEF's conceptual framework on the determinants of maternal and child nutrition, as described in last sentence of section “variable definition”.

We would like to add that the selection of the cut-off of 0.10 for the p value was to have a lenient criterion and achieve greater sensitivity to identify associated factors of interest for the occurrence of CAS. We used this cutoff point instead of 0.20 or 0.25 because the associated variables under study were previously identified based on the UNICEF conceptual model and the literature. Therefore, based on the theoretical model, it was possible to be more demanding for this cutoff, but at the same time not so restrictive (p < 0.05). In other words, given that the variable identification was carried out based on the conceptual framework, we decided on a more sensitive cut-off, but at the same time without increasing the type I error.

We added this sentences in line 222-226:

“The choice of a 0.10 threshold was made in consideration of the theoretical model guiding the identification of variables. This cutoff was selected to afford a more sensitive criterion while simultaneously avoiding an undue increase in the risk of type I error.”

6. **The authors built single construct of Concurrent Anemia and Stunting. Have you thought of considering testing a construct of determination of the outcome divided in four categories: only stunting, only anemia, CAS versus none? It would have been interesting to analyze and compare the single odds ratio of each outcome and both outcome to verify if the odds are higher when they are combined then only anemia or stunting.**

**Response**:

a) We really appreciate this comment. However, this approach and hypothesis suggested by the reviewer could matter of a new study. The objective and research question pre-specified in the research protocol considered as the outcome to be modeled the concurrence of anemia and chronic malnutrition, under a dichotomous measurement. This methodological approach has been used by the few studies that have addressed the same research question:

a. Nutr J. 2019;18: 10. doi:10.1186/s12937-019-0436-4,

b. Int J Environ Res Public Health. 2023;20: 6251. doi:10.3390/ijerph20136251,

c. Matern Child Nutr. 2018;14: e12597. doi:10.1111/mcn.12597,

d. Population Health 12 (2020) 100683.

**Reviewer #2:**

1. **Title: modify as: Concurrence of anemia and stunting and associated factors among children aged 6 to 59 months in Peru**

**Response:**

a. Thank you for this advice, we accept the recommendation.
2. **Lines # 23 and 24: bivariate analysis and multivariate analysis make it bivariable analysis and multivariable analysis. And also in entire your document.**

**Response:**

a. We reviewed articles published in Plos One and verified that the authors use without distinction the terms “multivariable” or “multivariate” and “bivariable” or “bivariate”

We decided to maintain the use of “multivariate” and “bivariate” analysis.

3. **Line #24, you said, “We applied a statistical criterion (p < 0.10 in the crude analysis)." Why did you use p<0.1? Why don't you use 0.2, 0.25, or 0.3?**

**Response**:

a. Thank you for the comment. This information belongs to abstract; we have a limited number of words to explain the statistical detail. We incorporated this clarification into the Statistical Analysis section of the paper in response to the first reviewer's fifth observation.
4. **To determine the height-for-age Z-score of the under-5 children, which software was used?**

**Response**:

a. Thank you for the comment. This information belongs to abstract; we do not have enough space to explain this particularly concern. We explain in variable definition section that “Stunting was defined when the child had a height-for-age Z-score less than minus two (-2) standard deviations (SD) below the median according to the 2006 WHO child growth standard (variable HW70)”. This data already exists in database of DHS 2022, this database is freely available in .sav extension. We do not directly calculate this z score.
5. **Lines 160 and 161 were categorized as not stunting (HW70 ≥ -2 SD), moderate stunting (-3 SD ≥ HW70 < -2 SD), and severe stunting (HW70 < -3 SD). Since you used binary logistic regression, it should be recategorized as a dichotomous variable.**

**Response:**

a. We really appreciate this comment. We add the text that explains this recategorization.
6. **Explain how Wealth index was analyzed**

**Response:**

a. Thank you for this request. In table 1. variable definition we present briefly the methodology used for the DHS program. This methodology uses the following definition: “The wealth index is a composite measure of a household's cumulative living standard. The wealth index is calculated using easy-to-collect data on a household's ownership of selected assets, such as televisions and bicycles; materials used for housing construction; and types of water access and sanitation facilities.”

We add this description in table 1 of supporting information.

7. **What is minimum dietary diversity according to this study?**

**Response:**

a. Our definition of minimum dietary diversity is described in table 1. This follows the methodology of DHS program. The text in table 1 of supporting information is: “Defined as "yes" according to DHS guidelines as consumption of at least 4 of 7 food groups during the day or night prior to the survey:

(a) Grains, roots and tubers, (b) Legumes and nuts, (c) Dairy products (milk, yogurt, cheese), (d) Meat foods (meat, fish, poultry and liver/vegetables), (e) Eggs, (f) Fruits and vegetables rich in vitamin A, (g) Other fruits and vegetables.

Otherwise, it was recategorized as "no".

8. **Line # 185: Table 1: Variable Definitions What is the importance of this table? If needed, you can submit it as supporting material alone.**

**Response:**

a. Thank you for the comment. This table permit us to give details to readers about definition of variables studied as potential associated factors to concurrent of anemia and stunting. We consider that this table is more efficient that text for describe theses definitions. We accept the suggestion of the reviewer and present the variable definition table as supporting material.
9. **The technique or method used to determine the concurrent of anemia and stunting is not clearly stated. Please state it clearly under data measurement or data analysis.**

**Response**:

a. Thank you for the comment. We explain this method in three first paragraphs of section: Variable definition. Also, more details about measurement during the primary survey (DHS-2022) can be found in section “Procedures for anthropometry and hemoglobin measurement”.
10. **Line #277-297: It is about bivariable analysis and table 4. What is the importance of describing bivariables in detail? It is better if you mention only the potential candidate of the variable that was selected for multivariable logistic regression.**

**Response**:

a. Thank you for the comment. We are agree with the reviewer. The section Bivariate Analysis was reduced, we eliminate most of the data reported in the table 3.
11. **Line # 302-310: Do you think that crude-level association is reported as a statistically associated variable?**

**Response:**

a. Thank you for your observation. We are afraid we misunderstood the comment. However, we would like to clarify that this section describes the findings of the logistic regression analysis and shows the measures of strength of association with CAS considering the simultaneous effect of the variables under study. This findings as those of model 2 and model 3 in section “multivariate analysis” are adjusted odds ratio and the respective p value. These are not crude associations.

Once again we thank you for taking the time to review our article and hope to meet your expectations.
